# Supplementary figures and images for: Acute and chronic effects of a light-activated FGF receptor in keratinocytes in vitro and in mice
Source: Life Sci Alliance. 2021 Sep 21;4(11):e202101100. doi: 10.26508/lsa.202101100 (PMC8473723; doi:10.26508/lsa.202101100)

1A

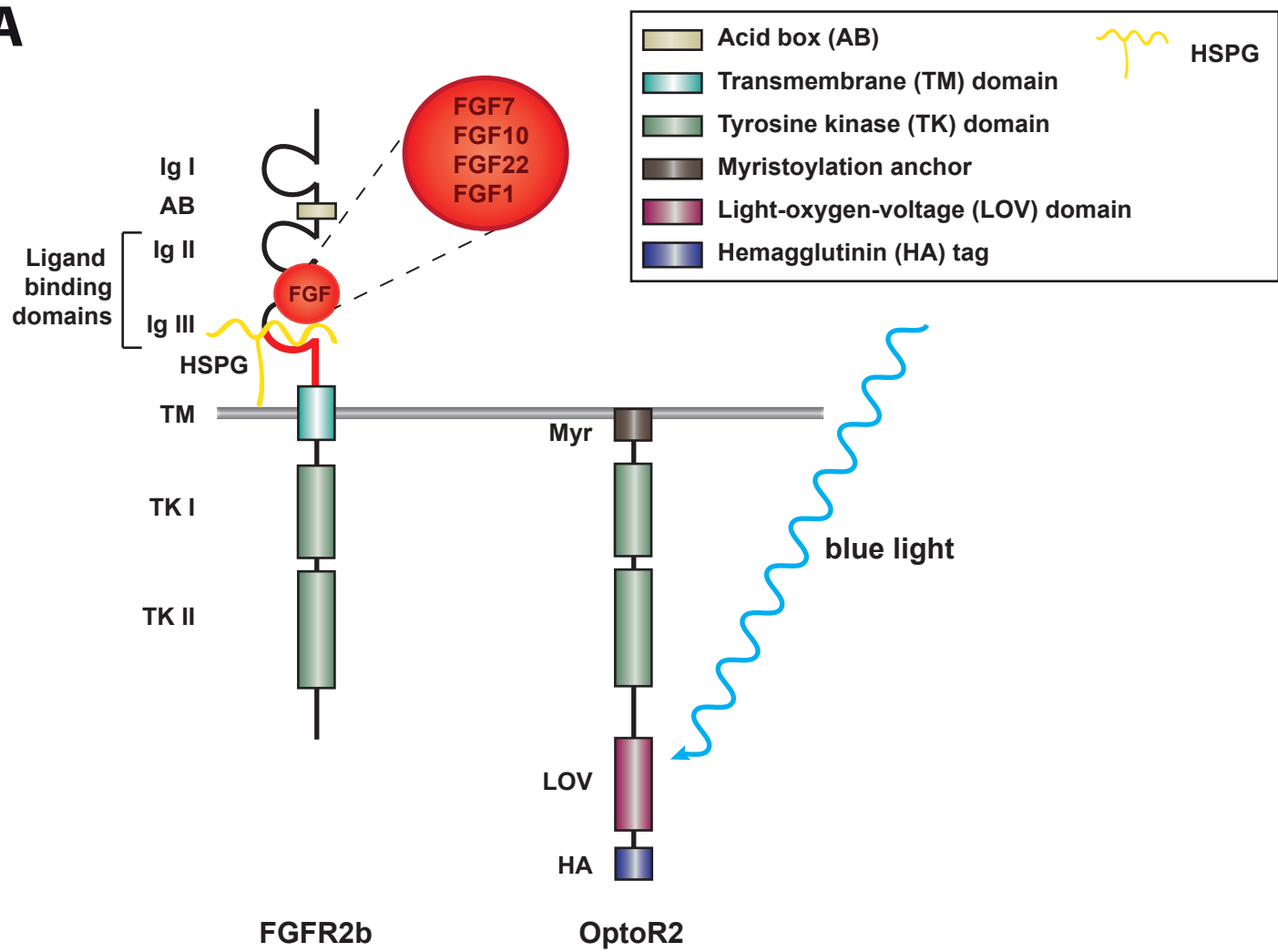

1B T125

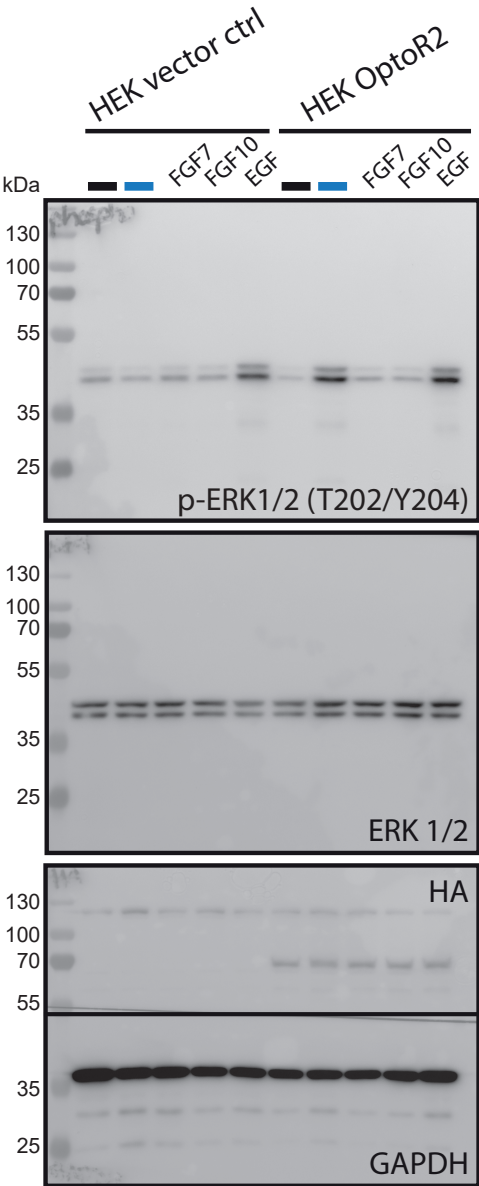

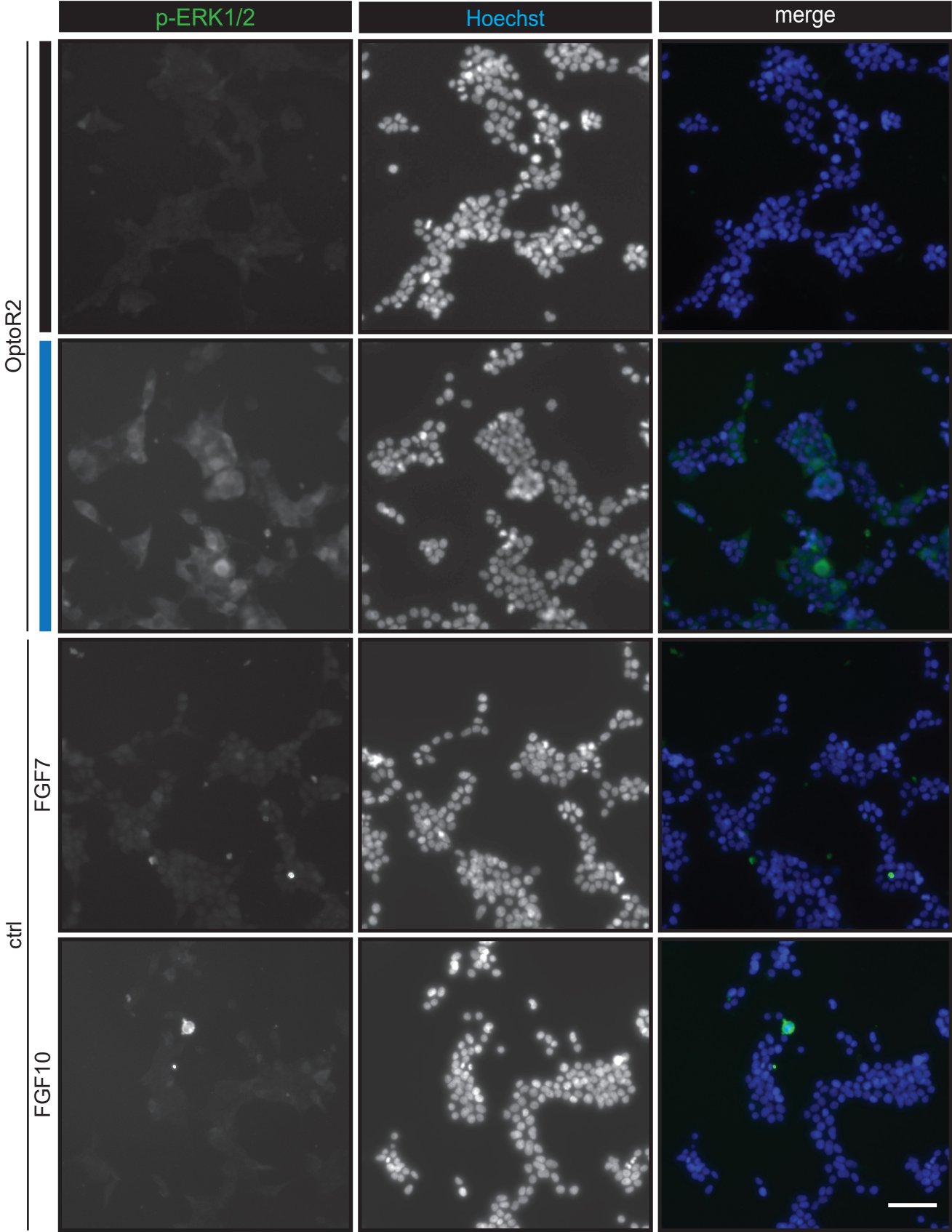

scale bar: 250  $\mu$ m

# 1D T120-T121

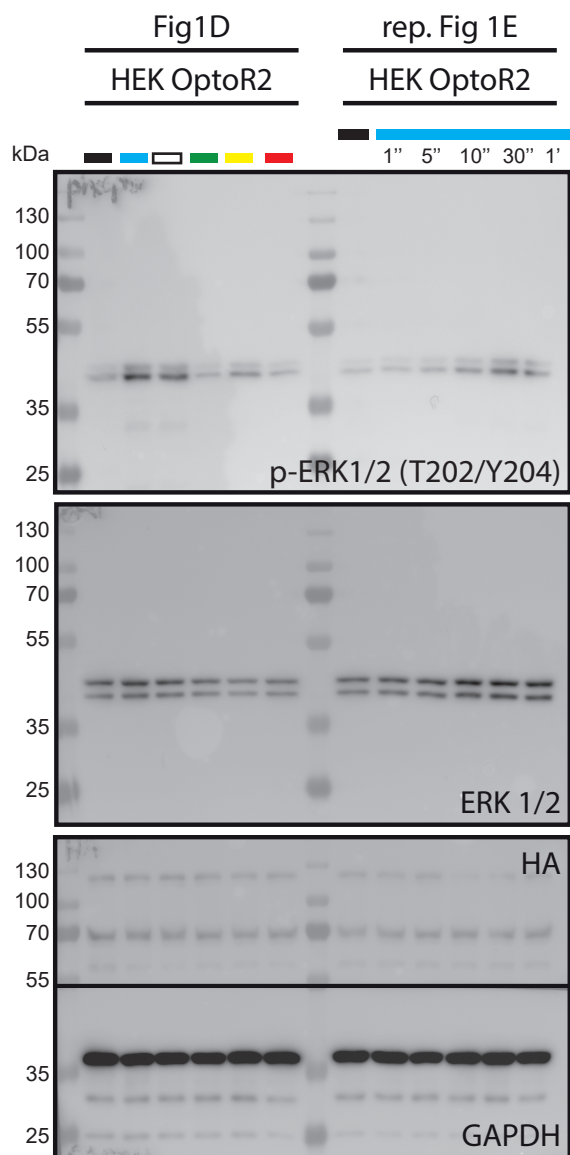

not included in the figure

**1E** SI8

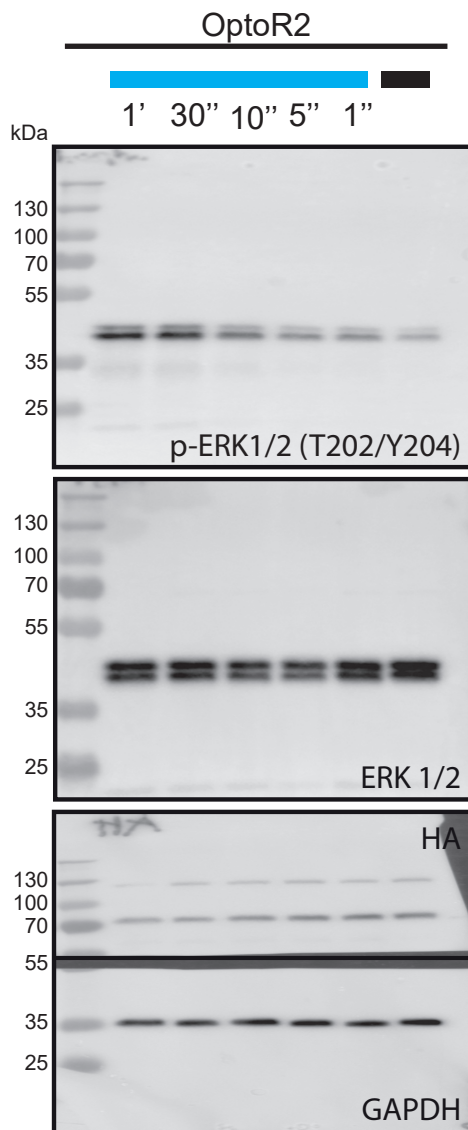

For Fig 1E, the blots were flipped vertically.

Supplement: Supplementary file 1 [file LSA-2021-01100_SdataF1.1.pdf]

# 3A mouse no 034

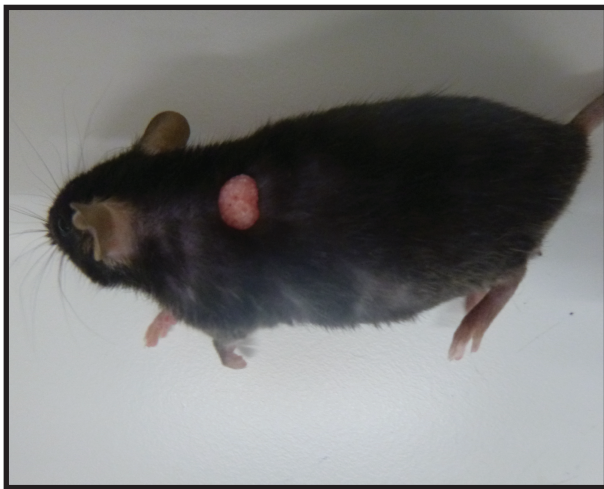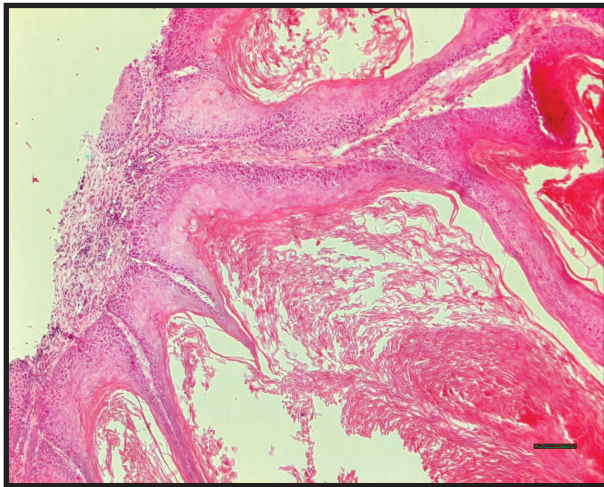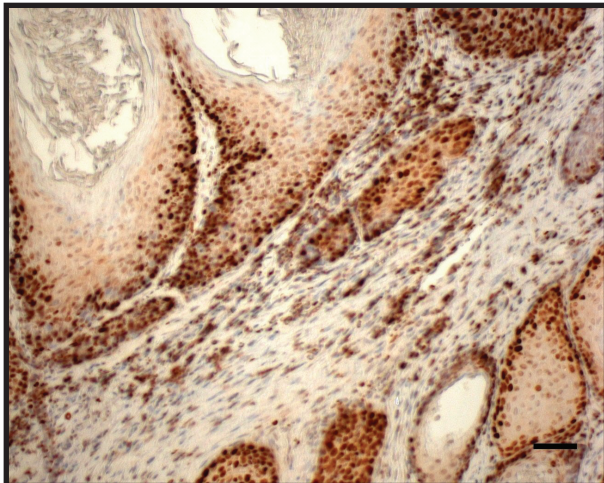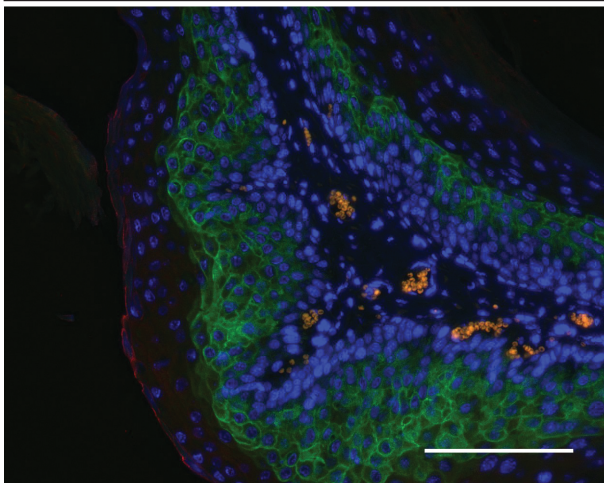

scale bar: 100  $\mu$ m

Supplement: Supplementary file 7 [file LSA-2021-01100_SdataF3.1.pdf]

# 5C T133

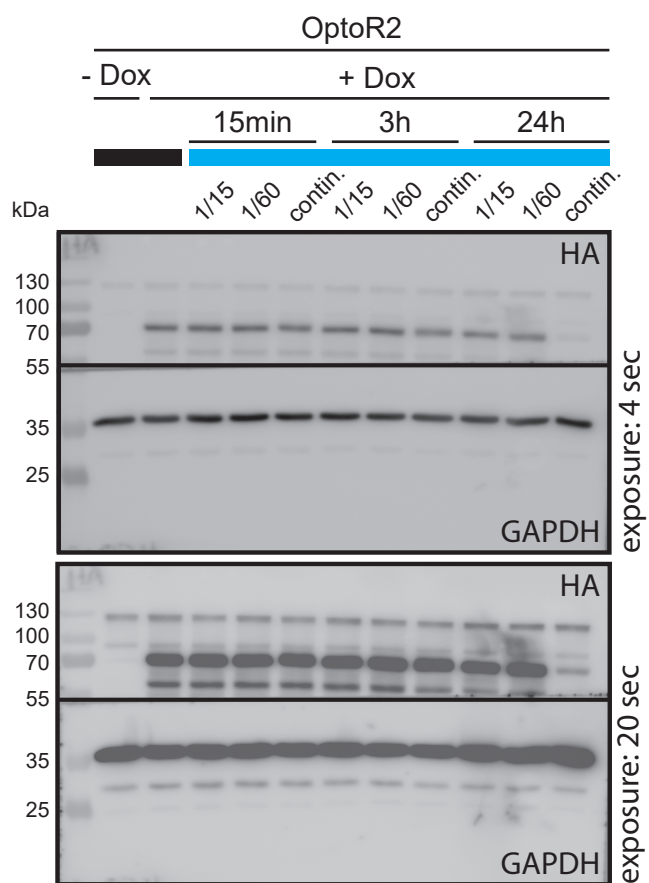

Supplement: Supplementary file 12 [file LSA-2021-01100_SdataF5.3.pdf]
