# Supplementary material for: Acute and chronic effects of a light-activated FGF receptor in keratinocytes in vitro and in mice
Source: Life Sci Alliance. 2021 Sep 21;4(11):e202101100. doi: 10.26508/lsa.202101100 (PMC8473723; doi:10.26508/lsa.202101100)

K14-OptoR2:

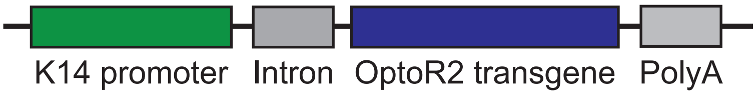

Dox-OptoR2:

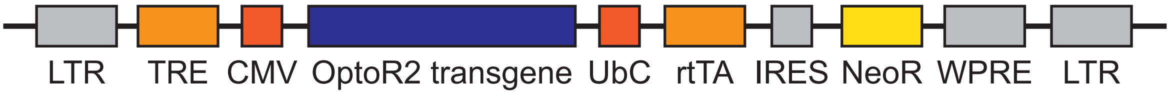

2B t=0: A1M; t=1.5years: S26

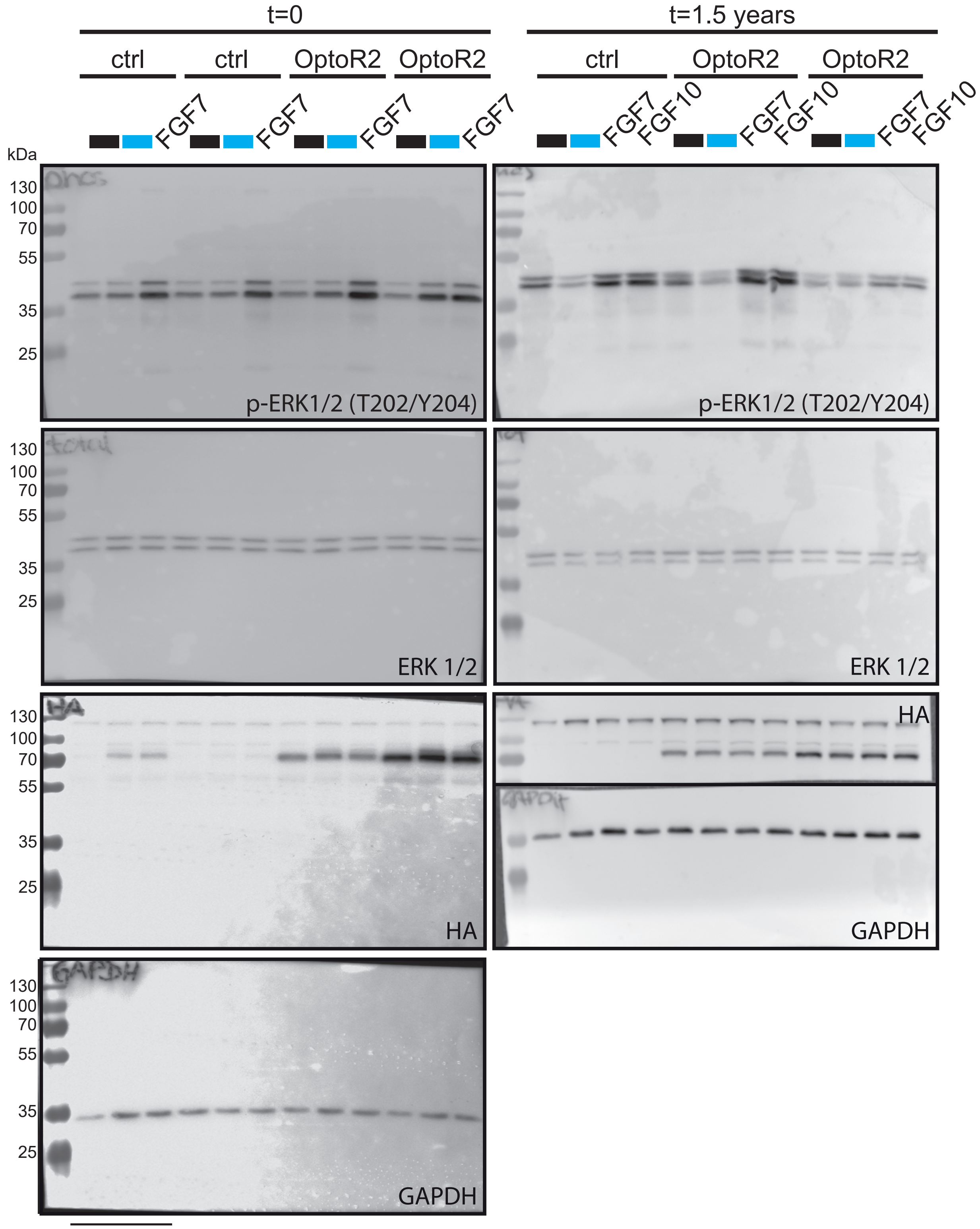

not included in the figure

2C S53

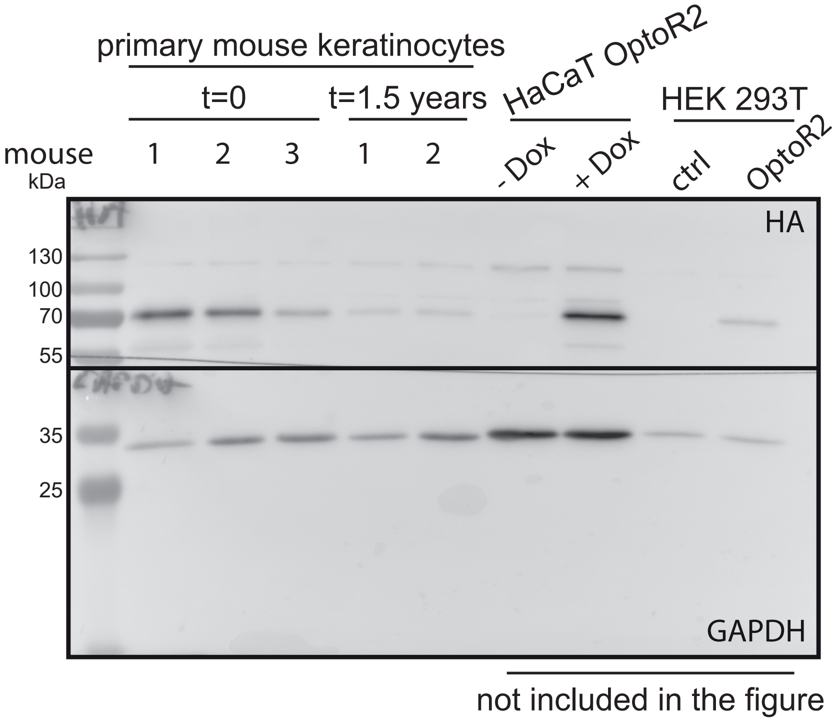

primary mouse keratinocytes
t=0
t=1.5 years
HaCaT OptoR2
HEK 293T
mouse
kDa
1
2
3
1
2
- Dox
+ Dox
ctrl
OptoR2
130
100
70
55
35
25
HA
GAPDH
not included in the figure

2D t=0:S1; t=6months: S37

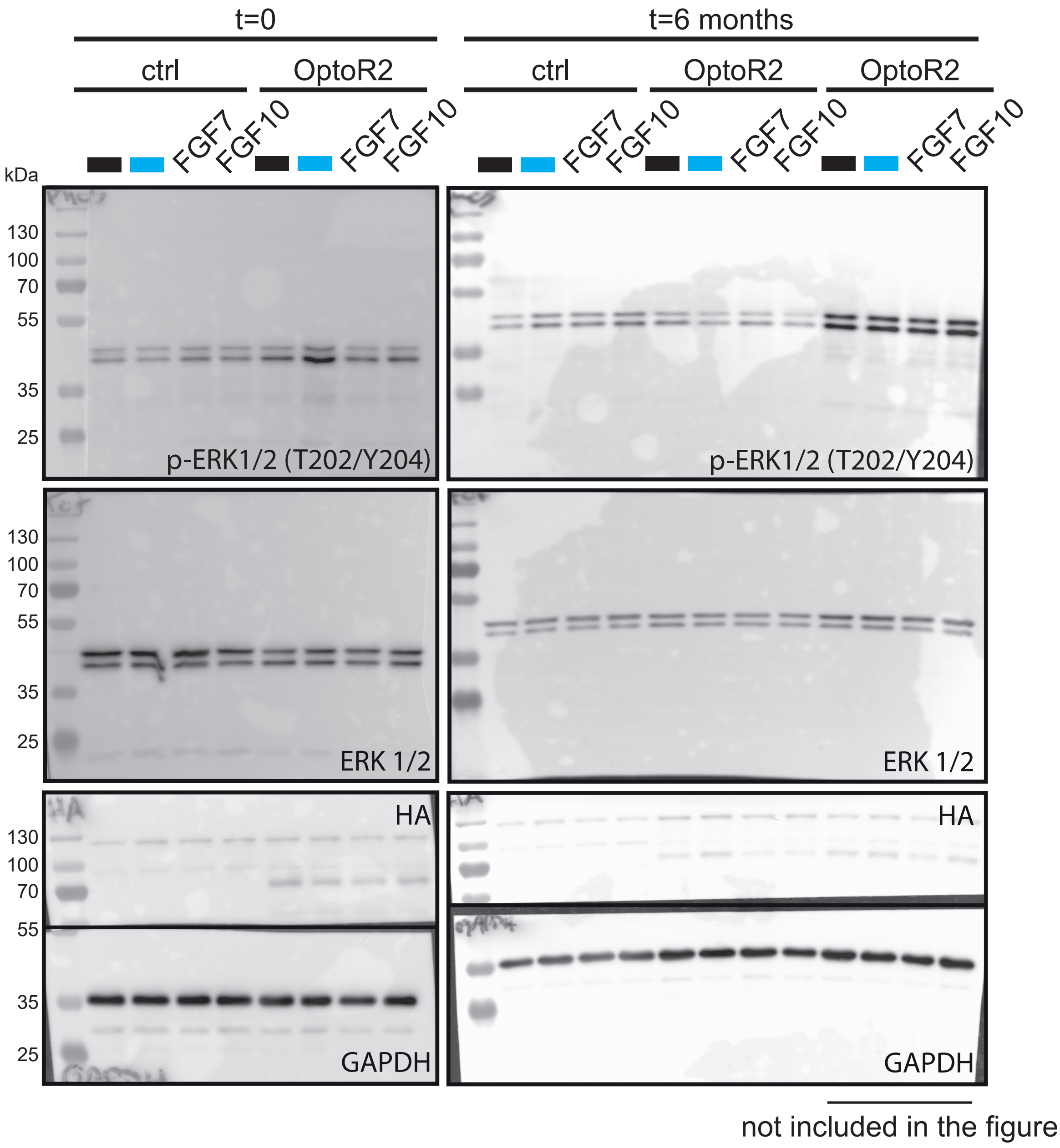

Supplement: Supplementary file 4 [file LSA-2021-01100_SdataF2.1.pdf]
