# Supplementary material for: Acute and chronic effects of a light-activated FGF receptor in keratinocytes in vitro and in mice
Source: Life Sci Alliance. 2021 Sep 21;4(11):e202101100. doi: 10.26508/lsa.202101100 (PMC8473723; doi:10.26508/lsa.202101100)
Supplement: Supplementary file 9 [file LSA-2021-01100_SdataF4.pdf]

4A T104

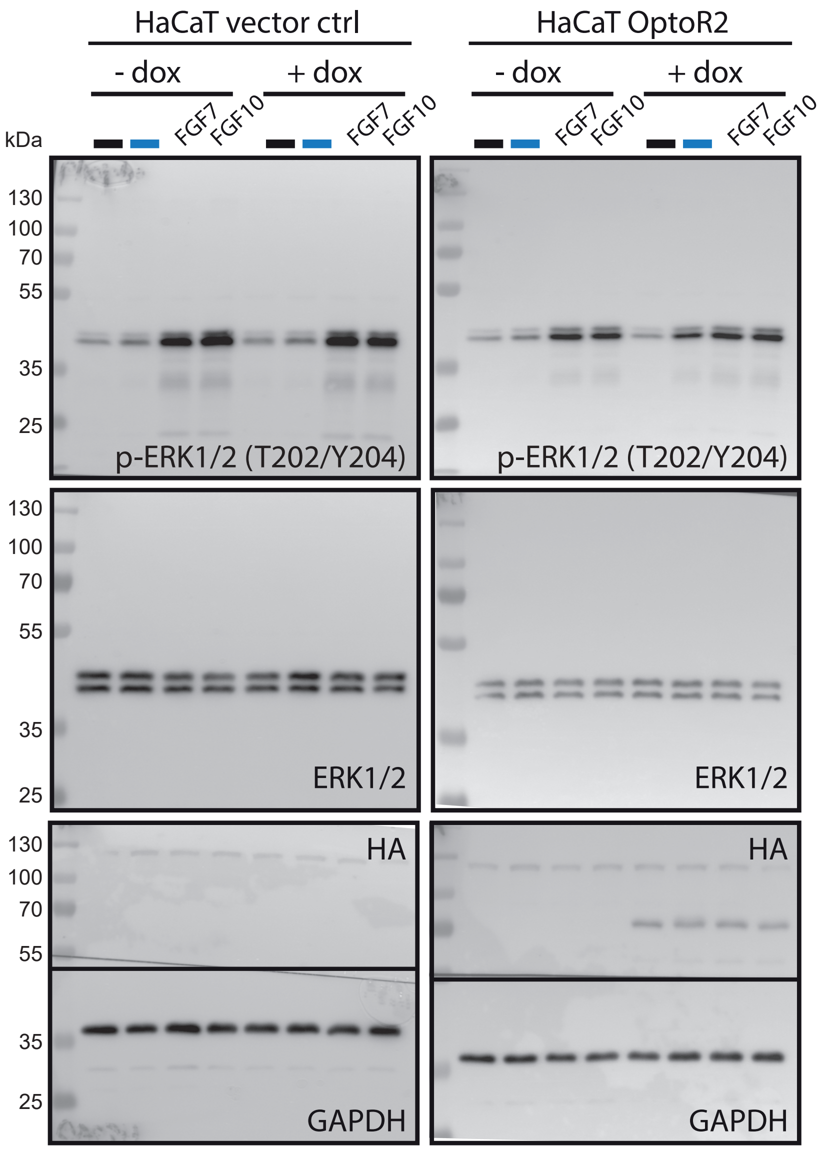
HaCaT vector ctrl
HaCaT OptoR2
- dox
+ dox
- dox
+ dox
FGF7
FGF10
FGF7
FGF10
FGF7
FGF10
FGF7
FGF10
kDa
130
100
70
55
35
25
p-ERK1/2 (T202/Y204)
p-ERK1/2 (T202/Y204)
130
100
70
55
35
25
ERK1/2
ERK1/2
130
100
70
55
35
25
HA
HA
GAPDH
GAPDH

4B T28

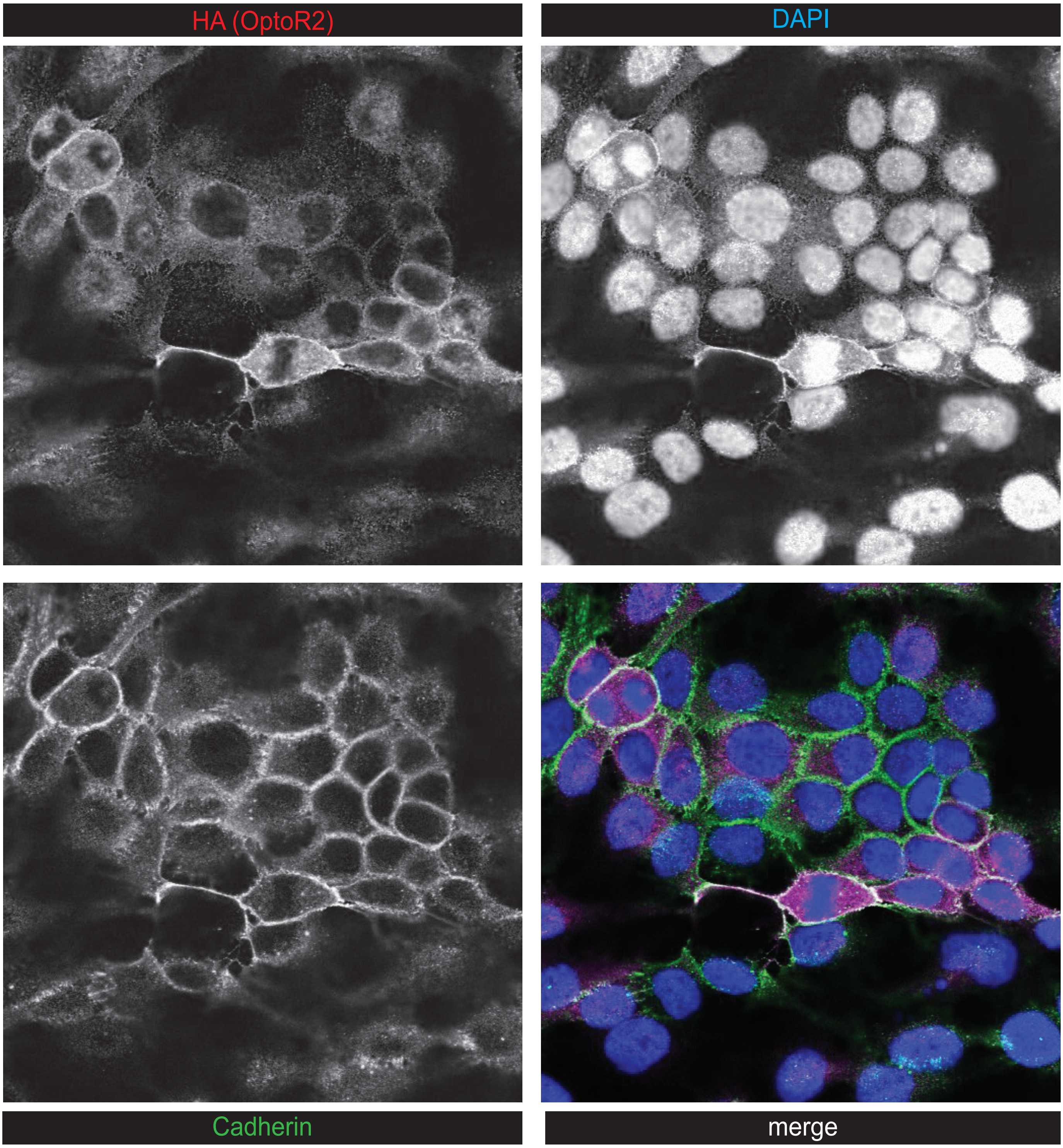

4C T92

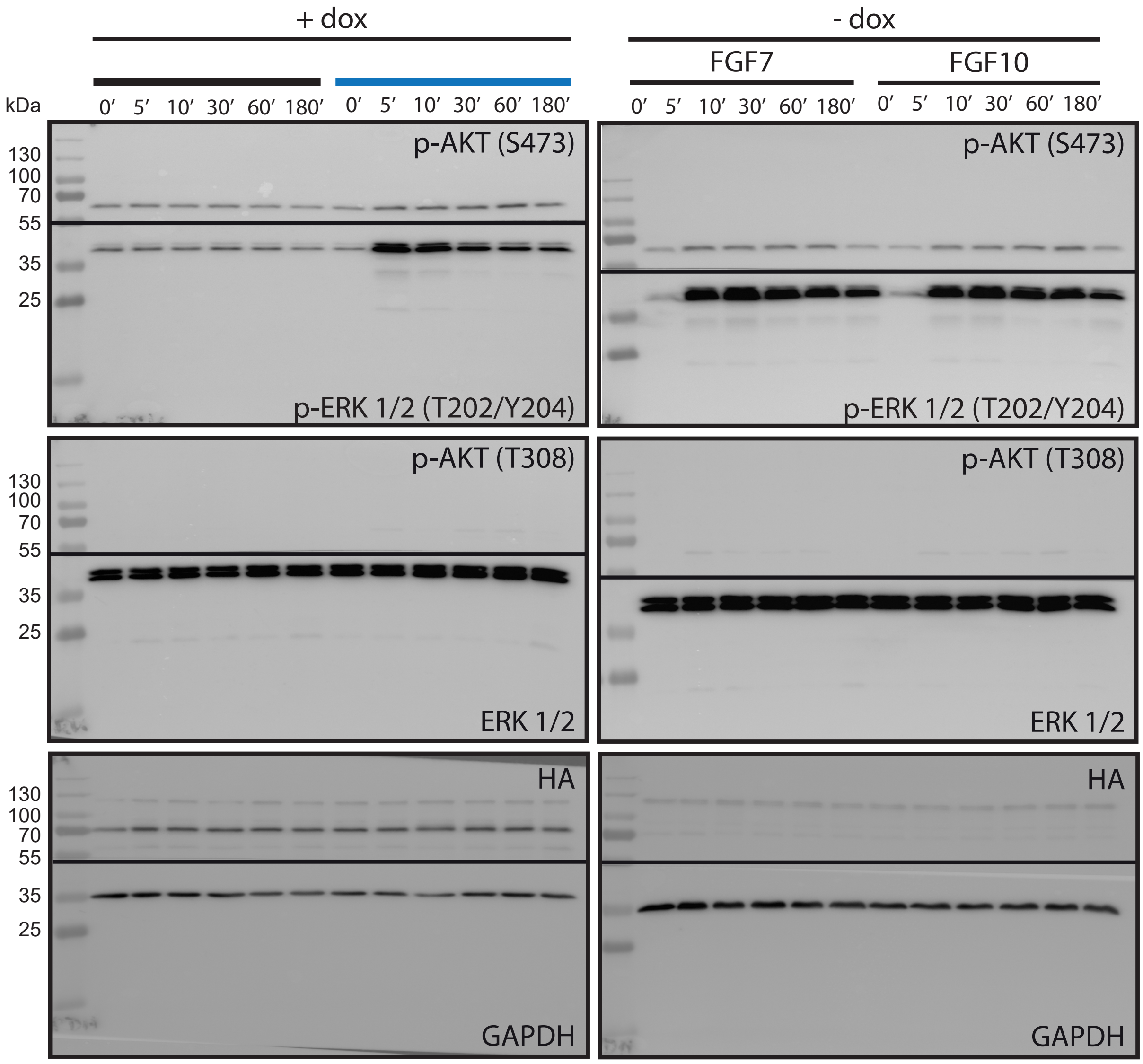

p-AKT(S473) and p-AKT (T308) are not included in the figure.

4C continued T92

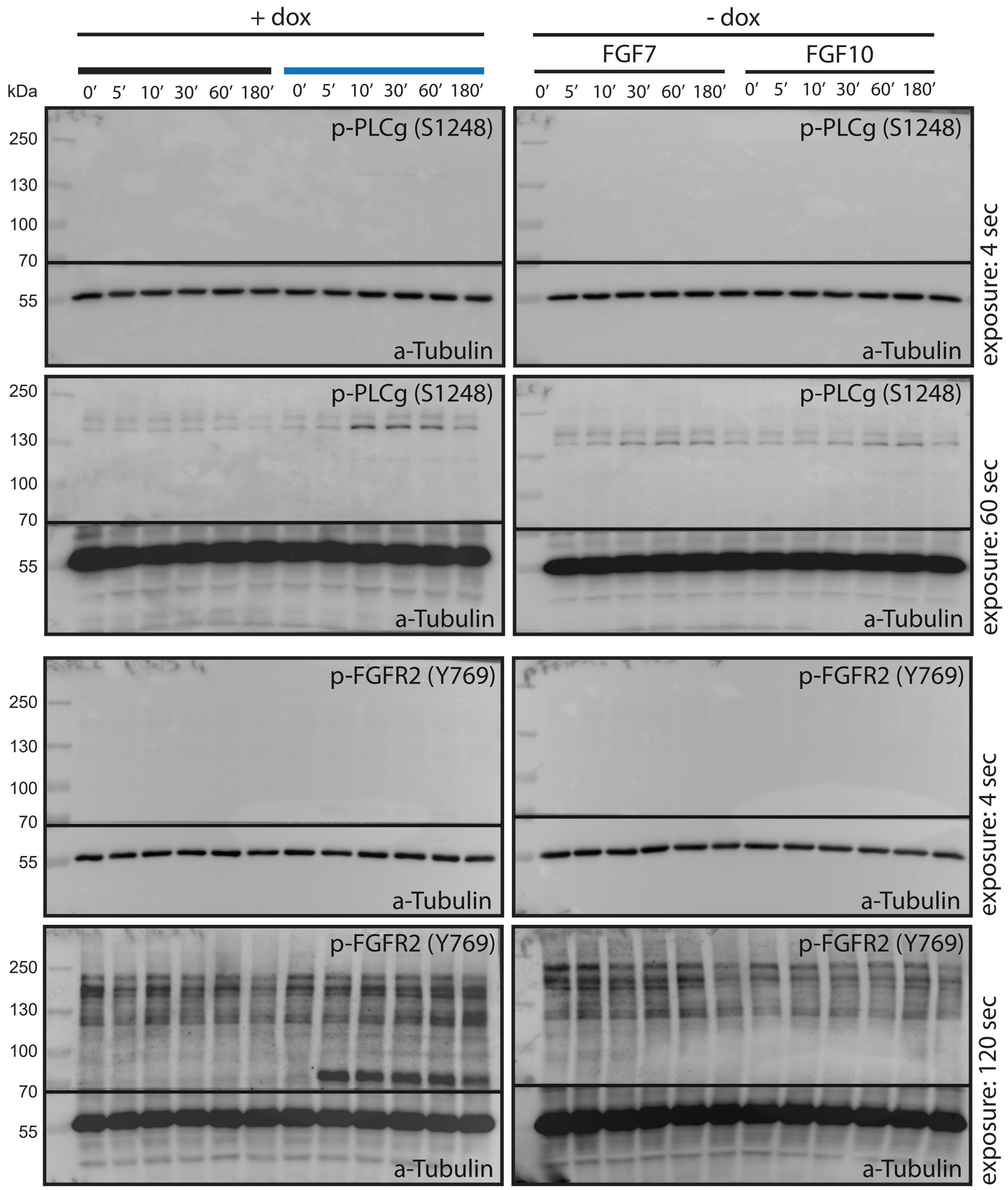

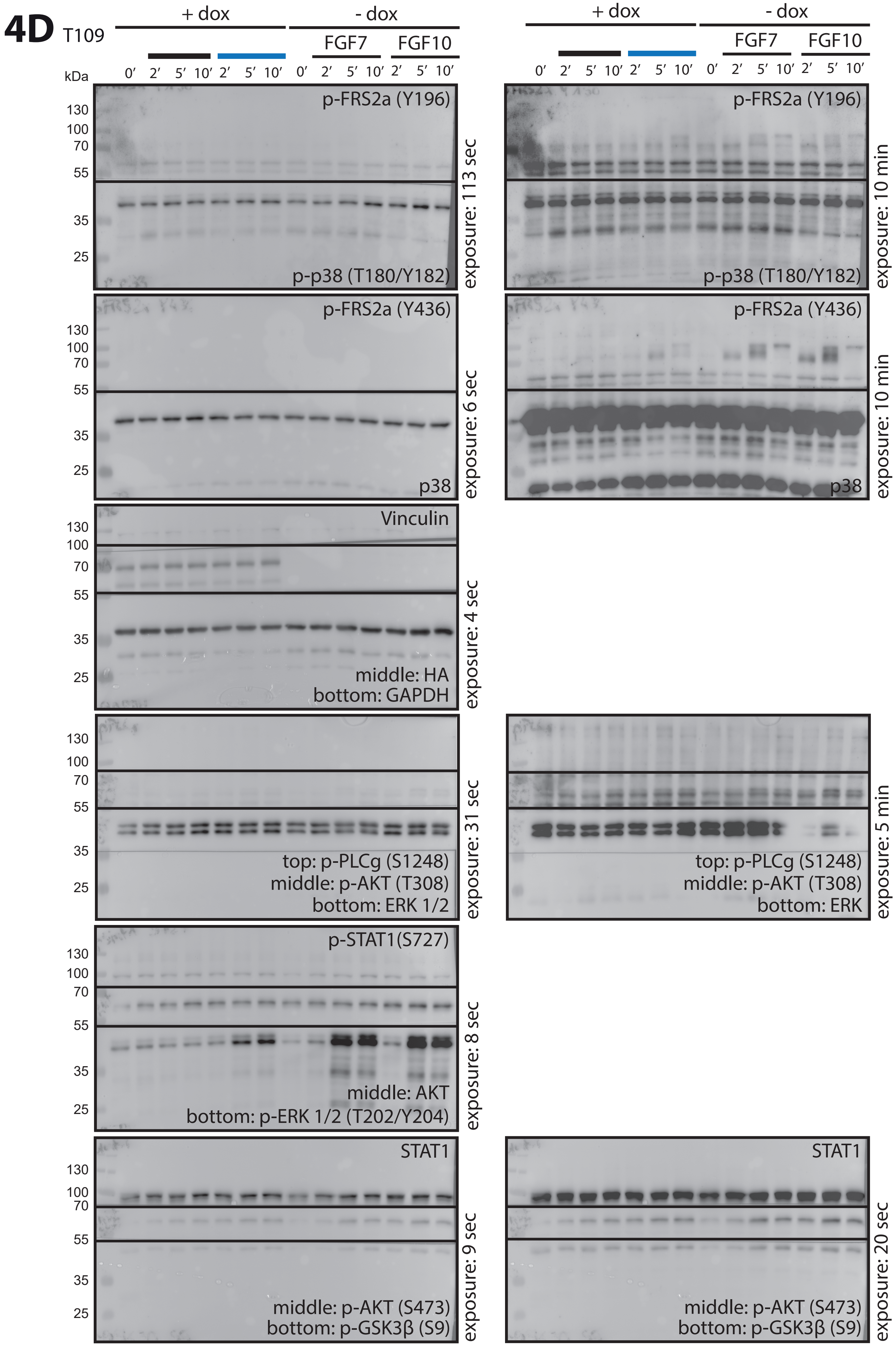

Vinculin, p-PLCγ (S1248), p-STAT1 (S727), STAT1 and p-GSK3β (S9) are not included in the figure.
